# Sandwich-type graphene electrochemical sensor for nucleic acid detection of SARS-CoV-2: Sandwich-type graphene electrochemical sensor

**DOI:** 10.3724/abbs.2025015

**Published:** 2025-03-11

**Authors:** Huan Yang, Yating Li, Donglin Cao, Li He, Yingjian Guo, Zhongming Liu, Haiyan Zhang

**Affiliations:** 1 General Hospital of Southern Theater Command Guangzhou 510010 China; 2 Guangzhou University of Chinese Medicine Guangzhou 510006 China; 3 The Affiliated Guangdong Second Provincial General Hospital of Jinan University Guangzhou 510317 China

Graphene and its derivatives exhibit excellent electrical and mechanical properties, including a high specific surface area, excellent electron mobility, and good biocompatibility, which make them ideal materials for fabricating biosensor devices [1]. Nevertheless, sensors based on pure graphene sensors still have certain limitations. For example, the number of dangling chemical bonds on the graphene surface is insufficient, which restricts the chemisorption of target molecules on the graphene surface. Additionally, graphene tends to stack and self-polymerize due to the presence of strong π-π interactions, van der Waals forces, and high surface energy, which leads to limitations in its semiconductor applications [2].

The incorporation of other nanomaterials ( *e*. *g*., metals, metal oxides, and conductive polymers) into graphene sheets has been demonstrated to prevent graphene agglomeration and improve the nanostructure [3]. Conductive polymers have been the subject of considerable interest within the context of electronic device manufacturing and the development of electrochemical sensors. This is due to a number of factors, including their low cost, simple preparation, high electrical conductivity, and high compatibility with modern electronic devices [4]. Polypyrrole (PPY), a widely used conductive polymer, has attracted attention, particularly in electrode modification. PPY exhibits excellent electrical conductivity, redox reversibility, biocompatibility, and environmental stability while also offering low production costs, making it an attractive option for use as a conductive polymer [5]. Concurrently, the distinctive structural characteristics of graphene and its oxides render them prospective conductive fillers for conductive polymers [6]. Consequently, the incorporation of graphene into polymers can compensate for their inherent limitations and enhance the long-term stability of sensing materials. The combination of graphene and conducting polymers represents a powerful means of preparing modified electrodes with good electrochemical properties, which have been successfully applied to the electrochemical detection of various biomolecules [7]. For example, Oliveira *et al* . [8] developed an electrochemical gene sensor based on PPY and graphene quantum dots for the detection of the PML/RARα fusion gene in childhood acute promyelocytic leukemia.

As a graphene derivative, reduced graphene oxide (rGO) is similar to graphene in numerous aspects, including favourable electrical conductivity, flexibility, low cytotoxicity, hydrophilicity, a substantial surface-area-to-volume ratio, and elevated chemical resistance. These attributes render rGO an exemplary matrix for nanocomposites [9]. Owing to the presence of hydrophilic and reactive functional groups, rGO is ideal for use in biosensors. The hydrophilic nature of rGO is instrumental in the assembly of biosensors, enabling the fabrication of sensing platforms through techniques such as drop-casting, spin-coating, ink-jet printing, and processing of electrode materials.

**Figure 1:**
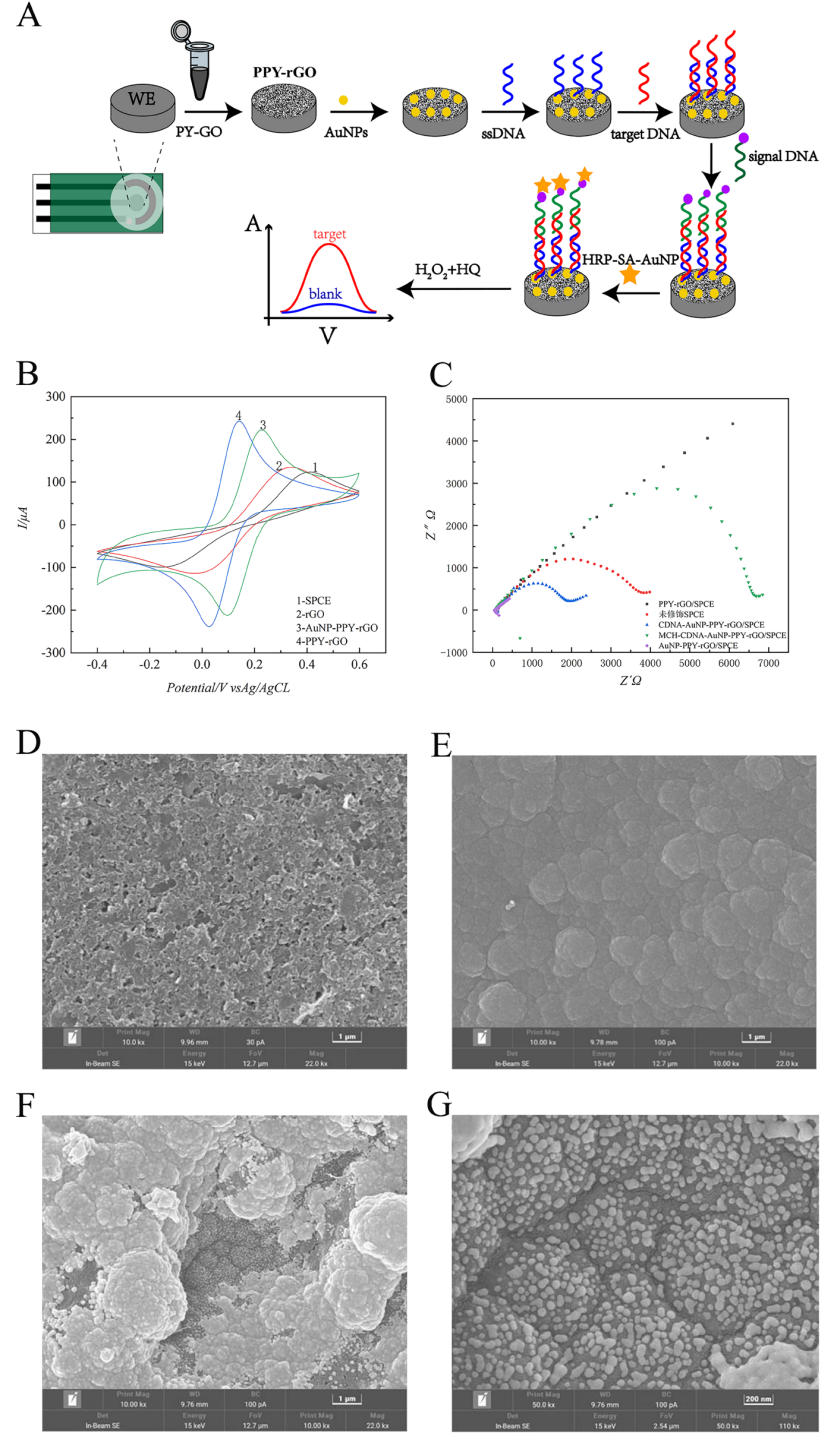
Figure 1 Establishment of the sandwich-type graphene electrochemical sensor (A) Schematic diagram of a sandwich-type electrochemical sensor based on PPY-rGO nanocomplexes. (B) CV detection of unmodified SPCE, PPY-rGO/SPCE, and AuNP-PPY-rGO/SPCE electrodes. (C) EIS analysis of the SPCE, PPY-rGO/SPCE, AuNP-PPY-rGO/SPCE, CDNA-AuNP-rGO/SPCE, and MCH-CDNA-PPY-AuNP-rGO/SPCE. (D–F) SEM images of unmodified SPCE (D), PPY-rGO/SPCE (E), and AuNP-PPY-rGO/SPCE (F). (G) AuNP-PPY-rGO/SPCE electrode surface with uniform deposition of AuNPs.

In the present study, a “sandwich” DNA hybridisation strategy was employed to construct an electrochemical DNA sensor based on PPY-rGO composite nanomaterials ( Figure 1A). PPY-rGO nanocomplexes were initially prepared by electrochemical deposition and subsequently modified on the surface of a screen-printed carbon electrode (SPCE) to increase the conductivity of the electrode, with SARS-CoV-2 serving as the target. The PPY-rGO nanocomplexes possess a substantial specific surface area and excellent conductivity, in addition to providing many attachment sites for the subsequent electrodeposition of AuNPs by cyclic voltammetry (CV). This enables the immobilization of a greater number of single-stranded DNA (ssDNA) probes, thereby enhancing the sensitivity and specificity of the sensor for the detection of target molecules. To further improve the specificity of detection, two DNA probes were designed on the basis of a sandwich hybridization strategy. One is a specific capture DNA (CDNA) with a sulfhydryl tag, and the other is a signal DNA (SDNA) with a biotin moiety, which can bind to horseradish peroxidase-streptavidin biofunctionalized gold nanoparticles (SA-HRP-AuNPs). Hybridization of the CDNA, target DNA (tDNA), and SDNA on the electrode surface formed a sandwich structure, whereby the SA-HRP-AuNPs bound to the biotin moiety. The detection of tDNA sequences was achieved via differential pulse voltammetry (DPV), which measures the current change of the sensor in hydrogen peroxide (H _2_O _2_) and hydroquinone (HQ) as the solvent electrochemical test solution. Electrochemical characterization and sensor performance testing were performed via a convenient electrochemical workstation and PSTrace software from PalmSens (Houten, Netherlands). SPCE electrodes were purchased from Changsha Sanjun Electronic Technology Co., Ltd. (Changsha, China).

The DNA oligonucleotide sequences used in this work were synthesized by Shanghai Sangon Biotechnology Co., Ltd. (Shanghai, China), and the detailed sequences are shown in Table 1. All the DNA oligonucleotide sequences were synthesized on the basis of the specific conserved regions of the SARS-CoV-2 N gene. The preparation of the DNA storage solution was as follows: the DNA lyophilization reagent was equilibrated to room temperature and then centrifuged at 4615 *g* for 30 s. Following centrifugation, the cap of the tube was carefully opened, and PBS buffer (0.01 M, pH 7.2) was added. The tube was subsequently placed in a vortex mixer and thoroughly agitated, after which the prepared DNA storage solution was dispensed and stored at –20°C.

**Table:** **Table 1** Artificial DNA sequences

Name	Sequence (5′→3′)
CDNA	SH-C6-ATGAGGAACGAGAAG
tDNA	CTTCTCGTTCCTCATCACGTAGTCGCAACA
SDNA	TGTTGCGACTACGTG-biotin
Triple-base mismatch sequence	CTTCACGTTTCTAATCACGTAGTCGCAACA
Non-complementary sequence	AGGAGTAGGAAGAGCTGTACCTGATTC CAG

The first step in the construction of the sensor was pre-treatment of the SPCE. The SPCE was subjected to an ultrasonic cleaning process in deionized water for a period of five minutes, after which it was dried under nitrogen. Thereafter, the SPCE was placed in 0.1 M PBS (0.1 M, pH 7.2) for activation. The electrode was subsequently activated by CV, with the number of scans set to 6, a scan voltage range of –1.5 to 0.6 V, and a scan rate of 0.1 V/s. Following activation, the electrode was rinsed and dried with ultrapure water and set aside.

The construction of the sensor involved modifying the material, immobilizing the probe and sealing the electrode surface. A solution of 0.1 M pyrrole monomer was prepared by dispersing the monomer in 0.1 M SDS, followed by ultrasonication for 15 min at room temperature. This process resulted in a solution of pyrrole monomer with uniform dispersion. The prepared 0.1 M PPY solution was used to dilute the rGO dispersion to 1 mg/mL, and the electrochemical deposition solution was obtained after ultrasonic dispersion to the homogeneous phase. The electrochemical deposition solution was then added to the surface of the SPCE, covering the surfaces of the three electrodes. The SPCE was modified by PPY-rGO nanocomposites via electrochemical deposition via CV, with a scan voltage range of 0.2–0.8 V, a scan rate of 0.05 V/s, and 10 cycles.

A chloroauric acid solution (0.2%) was then added dropwise to the surface of the SPCE, followed by electrochemical deposition via CV over a scan voltage range of –0.9 to 0.3 V with a scan rate of 0.1 V/s over 10 scans. The generation of AuNPs on the PPY-rGO surface served to increase the conductivity of the working electrode. Following electrodeposition, the electrodes were rinsed with ultrapure water and dried under nitrogen.

To improve the fixation efficiency of CDNA and prevent the formation of dimers of sulfur atoms at the extremities of the mercaptured DNA in solution, a 10 μM CDNA solution was mixed with a 10 mM TCEP solution at a 1:1 volumetric ratio. The solution was reduced for 60 min at room temperature, after which the CDNA was dissolved in 0.01 M PBS (pH 7.2) at a concentration of 1 μM. A total of 10 μL of the CDNA solution was added dropwise to the surface of the AuNPs-PPY-rGO-modified working electrode, resulting in firm immobilization of the sulfhydrylated CDNA on the surface of the working electrode via Au-S chemical bonding. The CDNA-AuNP-PPY-rGO/SPCE was obtained by incubating the components overnight (0.01 M, pH 7.2) to remove any unbound CDNA. To circumvent non-specific adsorption, 1 mM MCH was gradually introduced to the electrode surface, which was subsequently blocked at room temperature for 1 h. After being rinsed with PBS solution and dried, the sensor was successfully constructed.

The modified screen-printed electrode surfaces were characterized and analyzed via CV and electrochemical impedance spectroscopy (EIS). The electrochemical test mixture consisted of 5 mM [Fe(CN) _6_] ^3–/4–^ in 0.10 M KCl. As illustrated in Figure 1B, the PPY-rGO/SPCE-modified electrode exhibited a distinctive ferrocyanide redox peak, with a peak current that was markedly higher than that observed for the unmodified SPCE- and rGO-modified electrodes. Furthermore, the deposition of AuNPs on the PPY-rGO/SPCE-modified electrode resulted in a notable increase in the peak redox peak current, indicating that the PPY-rGO-modified electrode not only has excellent electrical conductivity, which facilitates electron transfer between the potassium ferrocyanide solution and the SPCE surface but also provides superior adsorption for the immobilization of AuNPs.

As shown in Figure 1C, the EIS characterization of the modified SPCE was consistent with the CV results. The radius of the curve in the EIS impedance profile represents the charge transfer resistance, which indicates that the surface of the unmodified SPCE had a high resistance. The transfer resistance of the electrode surface was significantly reduced after modification with PPY-rGO/SPCE, and its EIS impedance profile was close to a straight line. Following further modification by AuNPs deposition, the impedance spectrum presented as a straight line. However, following the immobilization of CDNA on the surface, an increase in the charge transfer resistance was observed. This was attributed to the phosphate backbone of the DNA molecules, which impeded electron transfer on the electrode surface. Furthermore, following confinement of the electrode surface using MCH, an additional increase in the charge transfer resistance was observed. This is attributed to the hindrance of electron transfer on the electrode surface by biomolecules.

To verify the successful modification of the AuNP-PPY-rGO nanocomposites on the electrode surface, the morphologies of the PPY-rGO- and AuNP-PPY-rGO-modified electrodes were characterized by a Tesken TESCAN MIRA field emission scanning electron microscope (SEM; Brno, Czech; Figure 1D). As shown in Figure 1E, many cauliflower-like PPY-rGO nanocomposites attached to the surface of the modified electrode. As clearly shown in Figure 1F, many AuNPs were highly distributed on the surface of the PPY-rGO nanocomposites with a coconut-like morphology. At higher magnifications, the SEM images revealed a large amount of uniformly deposited AuNPs, as shown in Figure 1G. SEM images of the modified electrode surface demonstrated that the PPY-rGO nanocomposites were successfully prepared via electrochemical deposition and stably modified on the surface of screen-printed carbon electrodes. Moreover, the PPY-rGO nanocomposites enhanced the electrocatalytic activity of the electrode surface and augmented the adhesion capacity of AuNPs on the electrode surface, thereby facilitating the successful immobilization of sulfhydrylated DNA probes.

**Figure 2:**
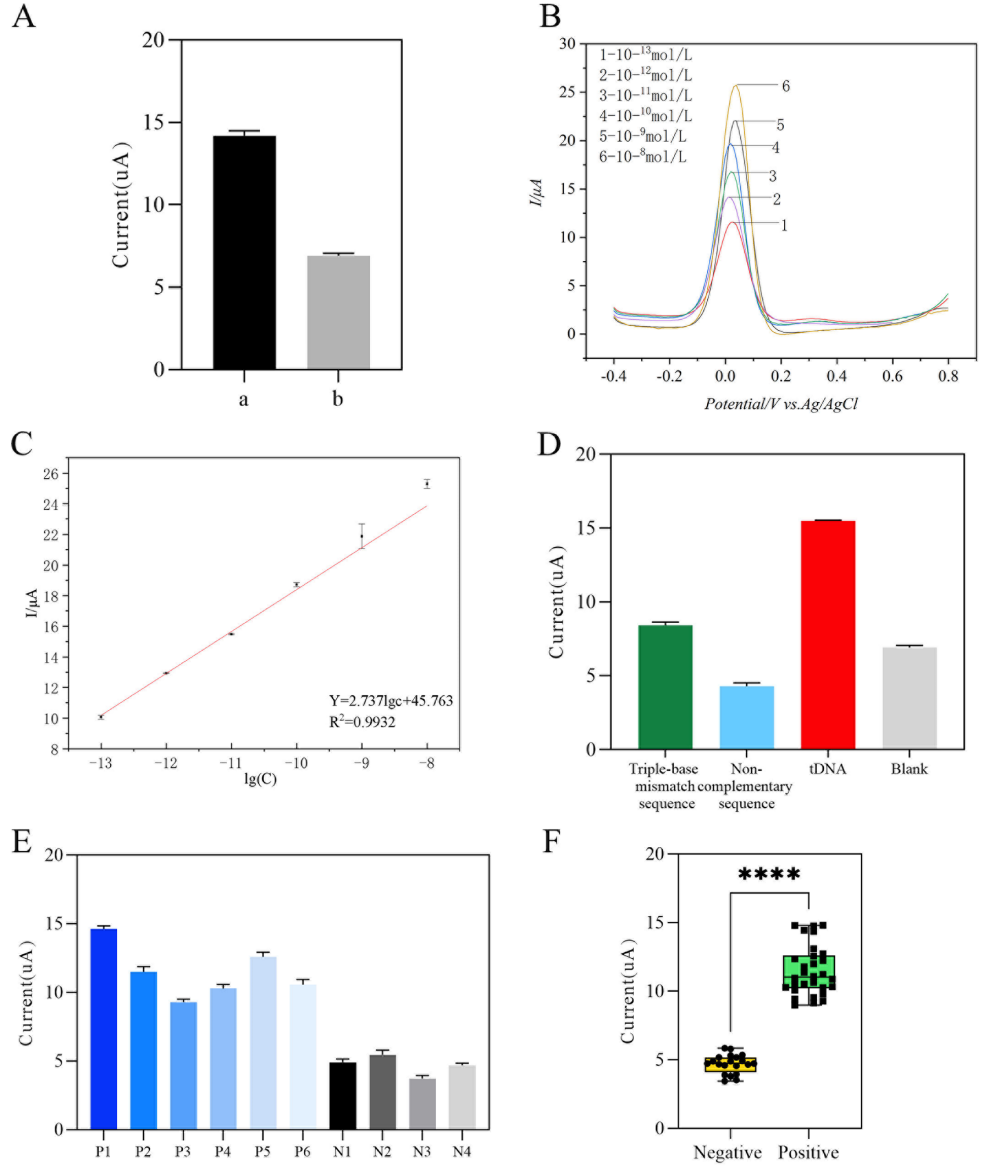
Figure 2 The AuNP-PPY-rGO/SPCE exhibits excellent detection performanc (A) Current response peaks of the AuNP-PPY-rGO/SPCE and MCH-DNA-AuNP-PPY-rGO/SPCE. (B,C) DPV curves after hybridization with different concentrations of tDNA (B) and linear fitting results (C). (D) DPV current response values (n = 5) for tDNA, triple-base mismatch sequences, and non-complementary sequences. (E,F) Current response of DPV (E) in positive samples and negative samples (n = 5) and significance analysis (F) of negative-positive samples (****P < 0.0001).

To ascertain whether the discrepancy between the electrodes created in the experiment could be employed in subsequent experiments, DPV was utilized to detect the current response peaks of eight AuNPs-PPY-rGO/SPCE and eight MCH-DNA-AuNP-PPY-rGO/SPCE in the electrochemical test solution (0.1 M KCl of 5 mM [Fe(CN) _6_] ^3–/4–^), with repeatability testing. As shown in Figure 2A, the AuNP-PPY-rGO/SPCE and MCH-DNA-AuNP-PPY-rGO/SPCE electrodes prepared via the same methodology demonstrated excellent repeatability and were suitable for further experiments.

The constructed electrochemical sensor is based on a sandwich-type DNA hybridization strategy, namely, CDNA, tDNA, and SDNA-specific hybridization. Initially, a series of tDNA solutions with varying concentrations (0.01 M PBS, pH 7.2) were prepared according to a specified concentration gradient. Subsequently, 10 μL of the tDNA solution to be tested was added to the electrode surface and hybridized at 37°C for 40 min. After hybridization, a PBS solution (0.01 M, pH 7.2) was used to rinse and remove any unbound DNA. SDNA (1 μM) was then added to the electrode surface and hybridized at 37°C for 30 min. The preparation of the SA-HRP-AuNP complex involved the centrifugation of 1 mL of the AuNP dispersion at 13,845 *g* for 15 min. Subsequently, 800 μL of the supernatant was aspirated and discarded, with the aim of further concentrating the gold nanoparticles. Subsequently, 20 μL of 10 μg/mL SA-HRP was mixed with the concentrated AuNPs and incubated at 4°C for approximately 12 h. Finally, the prepared SA-HRP-AuNP nanocomposite was added dropwise to the electrode surface at 37°C for 30 min. Following washing and drying, the electrochemical performance was evaluated via DPV, with the detection solution prepared in 5 mM H _2_O _2_ and 5 mM HQ solution. The DPV voltage range was set to ±0.4 V, the scan rate was 0.05 V/s, the pulse potential was 0.005 V, and the pulse time was 0.05 s.

The peak current gradually increased with increasing tDNA concentration ( Figure 2B). The linear fitting results are presented in Figure 2C, which demonstrate a strong linear relationship between the peak current of the sensor and the tDNA concentration in the solution, with a linear regression equation of IμA = 2.737lg(c) + 45.763. The overall fit was excellent, with a correlation coefficient of R ^2^ = 0.9932 and a good linear range of 10 ^–13^–10 ^–9^ M. The electrochemical sensor demonstrated satisfactory performance in the detection of SARS-CoV-2. In accordance with the limit of detection formula, LOD = 3σ/k (where σ is the standard deviation of the blank and k is the slope of the calibration graph), and LOD = 1.6 × 10 ^–14^ M for this sensor.

To verify the specificity of the electrochemical sensor based on the sandwich hybridization strategy constructed in the experiment, 10 ^–11^ M tDNA, a triple-base mismatch sequence, and non-complementary sequence were added dropwise to the surface of the modified electrode and hybridized at 37°C for 40 min. The electrochemical detection of the DPV was subsequently performed following binding with SDNA and SA-HRP-AuNPs. The results of the experiment are presented in Figure 2D. The peak current generated by the electrochemical sensor for detecting tDNA was significantly greater than that of the triple-base mismatch sequence and the non-complementary sequence. The peak current of the trinucleotide mismatch sequence was lower than that of the non-complementary sequence. This is because the three-base mismatch sequence and non-complementary sequence are unable to undergo complete hybridization with CDNA and SDNA. Therefore, the current on the electrode surface will not increase significantly. This result indicated that the interaction between CDNA and SDNA with mismatched target sequences was minimal.

In light of the aforementioned experimental results, an electrochemical sensor was deployed for the direct detection of SARS-CoV-2 in clinical samples. The clinical samples were obtained from the pharyngeal swabs collected by the Center for Disease Prevention and Control of the Southern Theatre Command. The center staff screened SARS-CoV-2-positive and -negative results via PCR. Clinical samples were stored in 1–3 mL of preservation solution at –80°C. Ten clinical throat swab samples of SARS-CoV-2 (inactivated by guanidine isothiocyanate) were detected by our sandwich-type graphene electrochemical sensor, of which six were SARS-CoV-2-positive throat swab samples, with cycle threshold (Ct) values less than 40. Four cases were negative throat swab samples, and the negative samples did not form a significant amplification curve in the PCR results or had a Ct value greater than 40. In accordance with the instructions provided by the manufacturer, sample RNA was extracted using the Tiangen kit (Shanghai, China). To prevent sample degradation or cross-contamination, it is essential to extract negative and positive samples in separate batches at different time and in different locations. The extracted nucleic acid samples were subsequently packaged and stored at –80°C. In the absence of any nucleic acid amplification process, 10 μL of the extracted RNA sample was added directly to the surface of MCH-CDNA-AuNP-PPY-rGO/SPCE. The average peak current was subsequently detected and recorded ( *n* = 5) via DPV following a 40-min hybridization period at 37°C. An independent sample *t* test with a confidence interval of 95% was employed for the statistical analysis.

Figure 2E shows the peak current of the positive samples (P1–P6) and negative samples (N1–N6). A *t* test revealed a significant difference ( *P <*0.0001), and there was a statistically significant difference in the mean peak current between the negative and positive samples ( Figure 2F). The peak current of the positive samples was significantly greater than that of the negative samples, indicating that the biosensor constructed in the experiment is capable of distinguishing SARS-CoV-2-positive samples from SARS-CoV-2-negative samples without the need for nucleic acid amplification.

The advantages of mass production, low cost, and simple operation of screen-printed electrodes were employed in the construction of an electrochemical detection method for SARS-CoV-2 detection based on graphene nanocomposites. In this study, two specific probes were designed on the basis of an electrochemical sensor modified with AuNP-PPY-rGO nanomaterials and the sandwich DNA hybridization strategy. The catalytic effect of SA-HRP-AuNPs was used to further amplify the electrochemical signals. The electrochemical sensor is capable of detecting SARS-CoV-2 in the range of 10 ^–13^–10 ^–9^ M. The electrochemical sensor demonstrated excellent specificity and was suitable for preliminary screening of SARS-CoV-2 clinical samples.

The potential for graphene electrochemical nucleic acid sensors to make significant contributions to clinical medical detection is considerable, given their high sensitivity, high selectivity, and rapid response time. Furthermore, as scientific and technological advancements continue, graphene electrochemical nucleic acid sensors are anticipated to facilitate the real-time, rapid, and straightforward detection of target biomolecules, extend clinical detection beyond professional laboratories to public settings, and offer technical support for the advancement of personalized medical and health management.
